# Association between Platelet Count and In-Hospital Mortality in Critical Patients with Multiple Myeloma: A Cohort Study

**DOI:** 10.1371/journal.pone.0323429

**Published:** 2025-06-05

**Authors:** Yan Zeng, Shisong You, Ruili Yuan, Shuhan Yue, Jingwei Zhang

**Affiliations:** 1 Department of Hematology, Chengdu Second People’s Hospital, Chendu, China; 2 Department of BIood Transfusion, Chengdu Second People’s Hospital, Chengdu, China; Sai Gosavi Specialty Clinic / Nano Hospitals Bangalore / Saraswati Specialty Clinic, INDIA

## Abstract

**Background:**

Multiple myeloma (MM) is a malignant blood disease characterized by the abnormal proliferation of immature plasma cells in the bone marrow. Changes in platelet counts (PLT) may significantly impact patient mortality. This study investigates the correlation between platelet counts and mortality rates in critically ill multiple myeloma patients admitted to the Intensive Care Unit (ICU).

**Methods:**

A retrospective cohort study was conducted with 242 patients diagnosed with MM. Data on platelet count(PLT), red blood cell count(RBC), serum calcium levels, International Normalized Ratio (INR), Sequential Organ Failure Assessment (SOFA) Score, Simplified Acute Physiology Score II (SAPS II), and comorbidities were collected. The study captured the highest and lowest values of laboratory data for patients during their ICU admission. Logistic regression analysis and smooth curve fitting technique were used for analysis. Subgroup analysis was applied to detect cross interactions. Sensitivity analysis was applied to detect consistency.

**Results:**

When the minimum PLT (PLT-min) were treated as a continuous variable at every 10 × 10^9^/L, the multivariate logistic regression analysis revealed that decreased PLT-min levels was an independent risk factor for mortality rate of critically ill patients with MM [Odds ratio (OR)=0.94, 95% confidence interval (95% CI): 0.89‑0.99, p = 0.023]. Additionally, when PLT-min levels were categorized into tertiles as <95 × 10^9^/L (group 1), 95–160 × 10^9^/L (group 2), and >160 × 10^9^/L (group 3), a decrease in PLT-min levels was also associated with an increasing trend in hospital mortality rate. Compared to group 1, the OR values of group 2 and group 3 were 0.40((95% CI:0.15–1.07, p = 0.069) and 0.30 (95% CI: 0.11‑0.82, p = 0.020). The relationship between PLT-min and in-hospital mortality was found to be nonlinear. Subgroup analysis showed no significant interactions. Similar results were obtained when analyzing the association of maximum PLT(PLT-max) and mortality rate.

**Conclusions:**

In MM patients in ICU, both minimum and maximum PLT were significantly associated with an increased risk of in-hospital mortality in critical ill patients with MM. These findings are important and warrant further investigation.

## Introduction

Multiple myeloma (MM) is a hematologic malignancy characterized by the clonal proliferation of plasma cells in the bone marrow, leading to the production of non-functional immunoglobulins and disruption of hematopoiesis. Complications of MM include anemia, hypercalcemia, renal dysfunction, and bone disease. Globally, the incidence of MM is increasing, ranking second only to Hodgkin’s lymphoma, with its frequency rising with age. In Europe, the annual incidence rate ranges from 4.5 to 6.0 per 100,000 population, accompanied by a mortality rate of 4.1 per 100,000. The widespread use of proteasome inhibitors, immunomodulatory agents, monoclonal antibodies, and autologous hematopoietic stem cell transplantation has significantly improved the overall survival of MM patients. Transplant-eligible patients now have a median overall survival (mOS) exceeding 12 years. However, a subset of patients does not benefit from novel treatments and autologous transplantation, facing an overall survival of less than 2 years [1,2]. Currently, the selection of treatment and prognosis assessment for MM patients heavily relies on staging systems such as the Durie-Salmon (DS) staging system [3], International Staging System (ISS) [4], and Revised International Staging System (R-ISS) [5]. The first two staging systems do not consider the genetic and molecular abnormalities of myeloma cells, posing challenges in accurately evaluating disease complexity and treatment response among patients. The revised R-ISS staging score addresses this gap by incorporating cytogenetic abnormalities and lactate dehydrogenase (LDH) levels, thus further stratifying patients to identify those at high risk of early death. However, factors like age, tumor microenvironment, and inflammatory immune response are not factored into these staging systems [5]. Moreover, standardized genetic testing techniques for cytogenetics are often lacking in clinical laboratories. Therefore, exploring simpler and more objective prognostic markers as supplementary tools for prognosis assessment is crucial to enhance risk stratification. This not only assists clinicians in determining treatment strategies but also plays a crucial role in achieving personalized therapy.

Platelets play vital physiological roles in the body, participating not only in hemostasis and clotting processes but also in inflammation and the tumor microenvironment. Activated platelets serve as indicators of inflammation, secreting multiple cytokines essential for tumor cell growth, including interleukin-6 (IL-6), vascular endothelial growth factor (VEGF), platelet-derived growth factor (PDGF), and transforming growth factor-beta (TGF-β). These factors influence the tumor microenvironment, promoting tumor cell proliferation, stimulating tumor angiogenesis, increasing tumor cell invasiveness, and facilitating immune evasion by tumor cells [6,7]. Variations in PLT and function are closely linked to the occurrence, progression, and adverse outcomes of various solid tumors [8,9]. In vitro studies have demonstrated that reductions in PLT or inhibition of platelet function can slow tumor growth [8,9]. However, in several solid tumors, including colorectal cancer, gastric cancer, and non-small cell lung cancer, elevated PLT have been correlated with poor prognosis [10,11]. In contrast, low platelet counts and an inverted platelet/lymphocyte ratio have been observed as adverse prognostic factors in patients with multiple myeloma (MM) [7]. This discrepancy between MM and other solid tumors may be attributed to the unique pathological characteristics of MM. As a malignancy originating in the bone marrow, MM disrupts the maturation of normal megakaryocytes and inhibits platelet production due to the tumor burden caused by the clonal proliferation of malignant plasma cells. Consequently, platelet reduction may serve as an indirect indicator of tumor burden. Furthermore, the compromised immune function within the aberrant tumor microenvironment may lead to decreased platelet production through the secretion of abnormal inflammatory cytokines. For instance, transforming growth factor-beta 1, secreted by malignant plasma cells and involved in plasma cell growth and survival, can inhibit the maturation of megakaryocyte colony-forming units, resulting in thrombocytopenia [12]. Patients with MM also exhibit abnormal platelet activation, functional abnormalities, and shortened platelet lifespan [13]. Additionally, chemotherapy agents employed in the treatment of MM may contribute to reductions in platelet count through bone marrow suppression. Several studies have indicated a correlation between platelet count and poor prognosis in MM [14,15]. Thus, it can be hypothesized that reductions in platelet count may reflect various aspects of MM prognosis.In recent years, neutrophil-to-lymphocyte ratio (NLR) and platelet-to-lymphocyte ratio (PLR) have been used as inflammatory indicators, as they are believed to be closely related to the inflammatory state in the tumor microenvironment. These ratios have attracted attention in the assessment of prognosis in multiple myeloma (MM) patients. Studies have shown that high NLR, PLR, and low platelets count may be associated with poor prognosis. However, most of these studies have included newly diagnosed MM patients, with outcome indicators primarily being overall survival (5-year or 2-year) or progression-free survival.[14,16–19]Meanwhile the data often come from baseline data of patients diagnosed at a single center [14,16–19].Although these studies suggest that lower platelet values may be associated with poor prognosis, they have not conducted independent analyses of the association between platelet values and poor prognosis. It is worth noting that the secondary immune deficiency caused by drug treatment, especially novel treatment methods, may lead to severe infections and even death, but there is still a lack of research on adverse prognoses in critically ill MM patients [20].Platelet count is an economical and convenient component of routine blood tests that offers insights into both the hematopoietic function of the bone marrow and tumor burden, as well as the interactions between the tumor and its microenvironment. However, there is a lack of sufficient data evidence regarding the correlation between platelet values during the progression phase of MM and poor prognosis in critically ill patients. We hope to scientifically analyze the independent association between platelet values and mortality rates of MM patients during ICU hospitalization through a retrospective cohort study.

## Materials and methods

### Population and data collection

The study utilized data from the Medical Information Mart for Intensive Care (MIMIC-IV 3.0 version), which provided information on over 70,000 patients admitted to Beth Israel Deaconess Medical Center’s ICUs in Boston, MA, from 2008 to 2019 [21]. Access to the database involved completing the “CITI Data or Specimens Only Research” training course on the National Institutes of Health website, with approval granted to extract data for research purposes (Certificate Number for Zhang: ID: 13813672). Inclusion criteria specified adult patients with multiple myeloma admitted to the ICU (age>=18 years), with multiple myeloma defined by ICD-9 codes 20300, 20301, and 20302 or ICD-10 codes C9000, C9001, and C9002 [21]. Demographics, comorbidities, and laboratory indicators were extracted from the MIMIC-IV database. We used Structured Query Language (SQL) with PostgreSQL (version 13.0) and Navicat software (version 16.0) to identify the cohort and extract the relevant clinical information [22].. Specifically, for clinical parameters with multiple outcomes during a patient’s hospitalization, only the highest and lowest outcome were included. The primary outcome studied was in-hospital mortality post-ICU admission. For variables with missing data of less than 10%, the median was used for continuous variables, and the mode was used for categorical variables to replace the missing values. Variables with missing data exceeding 10% were excluded from the analysis. according to the recommendation of the Strengthening the Reporting of Observational studies in Epidemiology (STROBE) statement [23].

### Statistical analysis

Continuous variables with a normal distribution were presented as mean ± standard deviation (SD), while those without a normal distribution were expressed as median [interquartile range (IQR)]. Baseline characteristics were evaluated using Student’s t-test, Wilcoxon rank sum test, and Chi-square test. A multivariate analysis (binary logistic regression) with adjustments for major covariates was conducted to explore the association between PLT and in-hospital mortality. PLT was treated as a continuous variable (per 10 units) and categorical variable based on quantiles. Four models were constructed: Model 1 did not adjust variables; Model 2 adjusted demographic characteristics Gender, Age; Model 3 added adjusted laboratory parameters RBC-min, calcium-max and INR-min on the basis of Model 2; Model 4 added SOFA score and SAPSII score on the basis of Model 3, adjusted for all covariates. Subgroup analyses were grouped based on demographic characteristics and key covariates including RBC, SOFA score, and SAPSII, and since all three covariates were continuous variables, they were grouped based on median. Covariates selection was based on significant covariates identified through univariate analysis and previous literature [24]. Restricted cubic spline models were employed to investigate potential nonlinear dose-response relationships between PLT and mortality [25]. Statistical analyses were performed using SPSS Version 26.0 (IBM Corp. USA), R 3.3.6 (http://www.R-project.org), and Free Statistics Version 1.5 [26]. P-value < 0.05 was considered statistically significant.

## Results

### Patient characteristics

**Fig 1:**
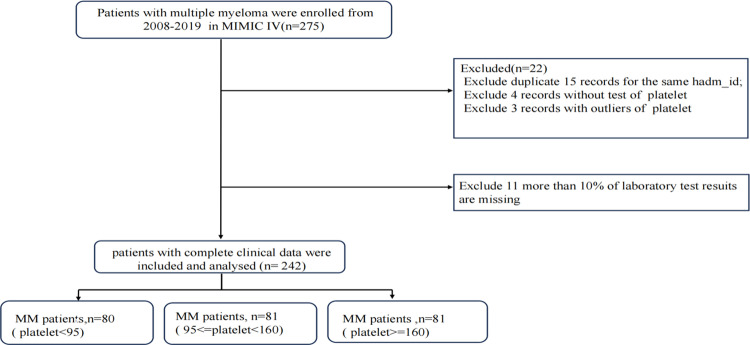
Flowchart of study population.

The flowchart outlining the inclusion and exclusion criteria is presented in Fig 1. Table 1 summarizes the baseline characteristics of the study participants, including demographic information, laboratory indicators and comorbidities. Participants were stratified into three groups based on the tertiles of the PLT- min. A total of 242 patients with MM were enrolled in this study, with an average age of 72.1 ± 11.4 years. Among the participants, 36.4% were male. 45 patients (18.59%) died during hospitalization. Significant differences were observed among the three groups regarding age, RBC count, PLT-min, white blood cell count (WBC), serum calcium, and SOFA score. However, no significant differences were identified in gender composition, other laboratory indicators, or comorbidities, including cardiovascular disease, kidney disease, diabetes, and liver disease (P > 0.05).

**Table 1:** Baseline demographic characteristics of the study population stratified by the lowest platelet count.

Variables	Total (n = 242)	Platelet-min quartiles (*10^9^/L)			*P* value
		Q1 (*n* = 81)	Q2 (*n* = 80)	Q3 (*n* = 81)	
		<95	95-160	>=160	
Gender, n (%)					0.743
Female	88 (36.4)	29 (35.8)	27 (33.8)	32 (39.5)	
Male	154 (63.6)	52 (64.2)	53 (66.2)	49 (60.5)	
Age	72.1 ± 11.4	69.5 ± 11.1	74.1 ± 11.0	72.7 ± 11.9	0.031
Mortality, n (%)	45 (18.6)	23 (28.4)	10 (12.5)	12 (14.8)	0.022
RBC-max(×10^12^/L)	5.3 ± 2.1	5.4 ± 2.2	5.0 ± 1.7	5.4 ± 2.4	0.326
RBC-min(×10^12^/L)	2.6 ± 1.1	2.4 ± 1.1	2.4 ± 0.8	2.9 ± 1.3	0.006
Hematocrit-min(%)	26.0 ± 5.8	23.3 ± 4.1	26.9 ± 5.9	28.0 ± 6.1	< 0.001
Hematocrit-max(%)	29.8 ± 5.6	27.9 ± 5.0	30.4 ± 5.0	31.3 ± 6.3	< 0.001
Hemoglobin-min(g/dL)	8.6 ± 1.9	7.8 ± 1.4	8.8 ± 1.9	9.2 ± 2.0	< 0.001
Hemoglobin-max(g/dL)	9.8 ± 1.8	9.3 ± 1.7	9.9 ± 1.6	10.1 ± 2.0	0.008
Platelets-min(×10^9^/L)	143.5 ± 105.6	47.4 ± 25.9	131.5 ± 20.7	251.5 ± 105.9	< 0.001
Platelets-max(×10^9^/L)	172.9 ± 118.4	71.5 ± 40.0	157.8 ± 33.8	289.3 ± 123.2	< 0.001
WBC-min(×10^9^/L)	6.1 (3.6, 9.5)	3.4 (1.6, 6.1)	6.4 (4.0, 8.7)	7.8 (5.5, 11.8)	< 0.001
WBC-max(×10^9^/L)	8.1 (5.0, 12.4)	5.1 (2.6, 9.1)	8.2 (6.1, 12.2)	10.3 (7.8, 15.9)	< 0.001
Urea nitrogen-min	27.5 (17.2, 46.0)	27.0 (17.0, 48.0)	28.5 (18.0, 39.2)	29.0 (17.0, 48.0)	0.975
Urea nitrogen-max	33.0 (20.0, 55.0)	30.0 (19.0, 55.0)	32.0 (20.8, 50.8)	36.0 (20.0, 56.0)	0.879
Calcium-min(mg/dL)	8.0 ± 1.1	7.8 ± 1.3	8.1 ± 1.0	8.2 ± 1.0	0.084
Calcium-max(mg/dL)	8.6 ± 1.2	8.3 ± 1.5	8.8 ± 1.0	8.6 ± 1.0	0.041
Chloride-min(mmol/L)	101.5 ± 7.4	103.7 ± 6.6	100.0 ± 8.2	100.7 ± 6.8	0.003
Chloride-max(mmol/L)	105.5 ± 6.8	107.6 ± 6.2	104.8 ± 6.7	104.0 ± 6.9	0.002
Creatinine-min(mg/dL)	1.3 (0.8, 2.4)	1.1 (0.8, 2.0)	1.4 (1.0, 2.4)	1.2 (0.8, 2.6)	0.326
Creatinine-max(mg/dL)	1.5 (1.0, 2.9)	1.5 (1.0, 2.1)	1.6 (1.2, 2.9)	1.5 (0.9, 3.0)	0.361
Glucose-min(mg/dL)	108.0 (91.0, 127.0)	108.0 (89.0, 129.0)	108.5 (93.0, 130.0)	107.0 (91.0, 126.0)	0.861
Glucose-max(mg/dL)	141.5 (116.0, 171.0)	142.0 (117.0, 171.0)	147.5 (117.8, 175.5)	131.0 (110.0, 160.0)	0.355
Sodium-min(mmol/L)	136.0 ± 6.9	136.8 ± 6.3	134.8 ± 8.2	136.4 ± 5.7	0.169
Sodium-max(mmol/L)	139.2 ± 5.4	140.1 ± 5.5	138.7 ± 5.6	138.9 ± 5.1	0.213
Potassium-min(mmol/L)	4.0 ± 0.6	3.9 ± 0.6	4.0 ± 0.7	4.0 ± 0.6	0.479
Potassium-max(mmol/L)	4.6 ± 1.0	4.5 ± 0.9	4.8 ± 1.1	4.6 ± 0.9	0.117
INR-min(s)	1.2 (1.1, 1.4)	1.3 (1.1, 1.5)	1.3 (1.2, 1.4)	1.2 (1.1, 1.4)	0.051
INR-max(s)	1.3 (1.2, 1.6)	1.4 (1.2, 1.8)	1.4 (1.2, 1.6)	1.2 (1.1, 1.5)	0.009
PT-min(s)	15.5 ± 6.1	15.2 ± 3.9	15.8 ± 5.6	15.5 ± 8.1	0.863
PT-max(s)	17.3 ± 7.9	17.5 ± 6.4	17.4 ± 7.0	16.9 ± 9.9	0.898
APTT-min(s)	28.9 (25.5, 33.9)	29.0 (25.3, 32.1)	28.6 (25.5, 36.3)	28.9 (25.9, 35.0)	0.675
APTT-max(s)	31.8 (27.5, 42.4)	31.6 (27.1, 38.0)	33.3 (27.6, 42.1)	30.7 (28.1, 45.7)	0.602
Cerebrovascular disease, n (%)					0.998
No	221 (91.3)	74 (91.4)	73 (91.2)	74 (91.4)	
Yes	21 (8.7)	7 (8.6)	7 (8.8)	7 (8.6)	
Pulmonary disease, n (%)					0.397
No	191 (78.9)	65 (80.2)	66 (82.5)	60 (74.1)	
Yes	51 (21.1)	16 (19.8)	14 (17.5)	21 (25.9)	
Diabetes, n (%)					0.321
No	217 (89.7)	71 (87.7)	70 (87.5)	76 (93.8)	
Yes	25 (10.3)	10 (12.3)	10 (12.5)	5 (6.2)	
Renal disease, n (%)					0.355
No	138 (57.0)	51 (63)	45 (56.2)	42 (51.9)	
Yes	104 (43.0)	30 (37)	35 (43.8)	39 (48.1)	
Liver disease, n (%)					0.125
No	236 (97.5)	77 (95.1)	78 (97.5)	81 (100)	
Yes	6 (2.5)	4 (4.9)	2 (2.5)	0 (0)	
SOFA score	3.6 ± 2.0	4.6 ± 2.5	3.1 ± 1.5	3.0 ± 1.4	< 0.001
SAPSII score	49.0 ± 13.1	51.0 ± 17.0	48.5 ± 9.8	47.4 ± 11.3	0.206

Abbreviations: SD = standard deviation; IQR = interquartile range; WBC = white blood cell; RBC = red blood cell; SOFA = Sequential Organ Failure Assessment; SAPS II = Simplified Acute Physiology Score II; min = the lowest value; max = the highest value

### Correlation analysis between variables and mortality rate

Table 2 presents the univariate analysis of risk factors associated with in-hospital mortality in these patients. The variables of RBC, PLT, serum calcium, INR, SOFA score, and SAPSII score show significant correlations with the mortality rate (P < 0.05). However, age, gender, and other comorbidities do not exhibit a correlation with the mortality rate.

**Table 2:** Association of covariates and in-hospital morality.

Variable	OR 95%CI	*p*
Gender	0.74 (0.38 ~ 1.43)	0.366
Age	1.02 (0.99 ~ 1.06)	0.111
RBC-max	1.3 (1.13 ~ 1.5)	<0.001
RBC-min	1.78 (1.31 ~ 2.41)	<0.001
Hematocrit-min	1.01 (0.95 ~ 1.06)	0.83
Hematocrit-max	1 (0.94 ~ 1.06)	0.936
Hemoglobin-min	0.99 (0.84 ~ 1.18)	0.948
Hemoglobin-max	0.99 (0.83 ~ 1.19)	0.923
Platelets-min	0.99 (0.99 ~ 1)	0.007
Platelets-max	0.99 (0.99 ~ 1)	0.004
WBC-min	1.02 (0.97 ~ 1.08)	0.362
WBC-max	1.01 (0.97 ~ 1.05)	0.53
Urea nitrogen-min	1.01 (1 ~ 1.02)	0.044
Urea nitrogen-max	1.01 (1 ~ 1.02)	0.136
Calcium-min	0.68 (0.49 ~ 0.95)	0.022
Calcium-max	0.78 (0.56 ~ 1.09)	0.14
Chloride-min	0.99 (0.95 ~ 1.03)	0.591
Chloride-max	1.01 (0.97 ~ 1.06)	0.571
Creatinine-min	1.05 (0.91 ~ 1.2)	0.524
Creatinine-max	1.05 (0.95 ~ 1.16)	0.363
Glucose-min	1 (0.99 ~ 1.01)	0.585
Glucose-max	1 (1 ~ 1)	0.041
Sodium-min	0.96 (0.92 ~ 1.01)	0.09
Sodium-max	1.01 (0.95 ~ 1.07)	0.697
Potassium-min	1.35 (0.8 ~ 2.28)	0.269
Potassium-max	1.11 (0.8 ~ 1.54)	0.52
INR-min	1.79 (1.11 ~ 2.9)	0.017
INR-max	1.51 (1.04 ~ 2.2)	0.03
PT-min	1.06 (1.01 ~ 1.11)	0.015
PT-max	1.04 (1 ~ 1.08)	0.036
APTT-min	1.03 (1.01 ~ 1.06)	0.011
APTT-max	1.01 (1 ~ 1.02)	0.079
Cerebrovascular disease	1.03 (0.33 ~ 3.23)	0.956
Pulmonary disease	0.77 (0.34 ~ 1.79)	0.549
Diabetes	0.82 (0.27 ~ 2.51)	0.725
Renal disease	0.96 (0.5 ~ 1.85)	0.91
Liver disease	4.62 (0.9 ~ 23.69)	0.067
SOFA score	1.2 (1.04 ~ 1.39)	0.015
SAPSII	1.06 (1.04 ~ 1.08)	<0.001

Abbreviations: RBC = red blood cell; WBC = white blood cell; INR = International Normalized Ratio; PT = Prothrombin Time; APTT = Activated Partial Thromboplastin Time; SOFA = Sequential Organ Failure Assessment; SAPS II = Simplified Acute Physiology Score II; min = the lowest value; max = the highest value.

In the multivariable logistic regression analysis (Table 3), the relationship between PLT-min (adjusted per 10 × 10^9^/L) and the mortality rate demonstrates statistical differences (OR 0.94; 95%CI 0.89–0.99, p = 0.023) adjusted for gender, age, RBC, serum calcium, INR, SOFA score, and SAPSII score. As the PLT-min in this study is relatively lower compared to the normal population, we divided it into three subgroups based on tertiles. When treated as a categorical variable, both the second group with platelet levels ranging from 95–160 × 10^9^/L and the third group with platelet levels ≥160 × 10^9^/L exhibit a significantly reduced risk of in-hospital death (OR: 0.36; 95% CI: 0.16–0.82; P = 0.015 & OR: 0.44; 95% CI: 0.20–0.96; P = 0.038). The first group, with a platelet count <95 x10^9^/L, serves as the baseline reference. In model 2, after adjusting for age and gender, the platelet count, whether treated as a continuous or categorical variable, remains significantly associated with an increased risk of in-hospital death. Further adjustments for RBC, serum calcium, and INR levels in model 3 reveal that the PLT-min continues to be significantly associated with an increased risk of mortality. Model 4, incorporating SAPSII and SOFA scores in addition to model 3, indicates a significant association between the PLT-min as a continuous variable and the risk of in-hospital mortality. When treated as a categorical variable, it still demonstrates a trend correlation, although the statistical significance only appears in the third group(p = 0.020).

**Table 3:** Multivariable logistic regression analyses of the association of PLT-min with in-hospital mortality.

	Platelet-min		Platelet-min quartiles				
	Total N = 242,n.event% = 45(18.6%)		Q1(N = 81);n.event% = 23(28.4%)	Q2(N = 80);n.event% = 10(12.5%)		Q3(N = 81);n.event% = 12(14.8%)	
	OR (95%CI)	*p*	OR (95%CI)	OR (95%CI)	*p*	OR (95%CI)	*p*
Model1	0.94 (0.90-0.98)	0.007	1(Ref)	0.36 (0.16-0.82)	0.015	0.44 (0.20-0.96)	0.038
Model2	0.93 (0.89-0.97)	0.002	1(Ref)	0.30 (0.13-0.71)	0.006	0.38 (0.17-0.85)	0.018
Model3	0.93 (0.89-0.98)	0.008	1(Ref)	0.37 (0.15-0.91)	0.030	0.27 (0.11-0.67)	0.005
Model4	0.94 (0.89-0.99)	0.023	1(Ref)	0.40 (0.15-1.07)	0.069	0.30 (0.11-0.82)	0.020

The lowest platelet count was entered as continuous variable per 10 × 10^9^ Model1 crude model Model2 adjusted for Gender, Age. Model3 adjusted for Model1 and RBC-min,calcium-max, INR-min Model4 adjusted for Model2 and SOFA score, SAPSII.

**Fig 2:**
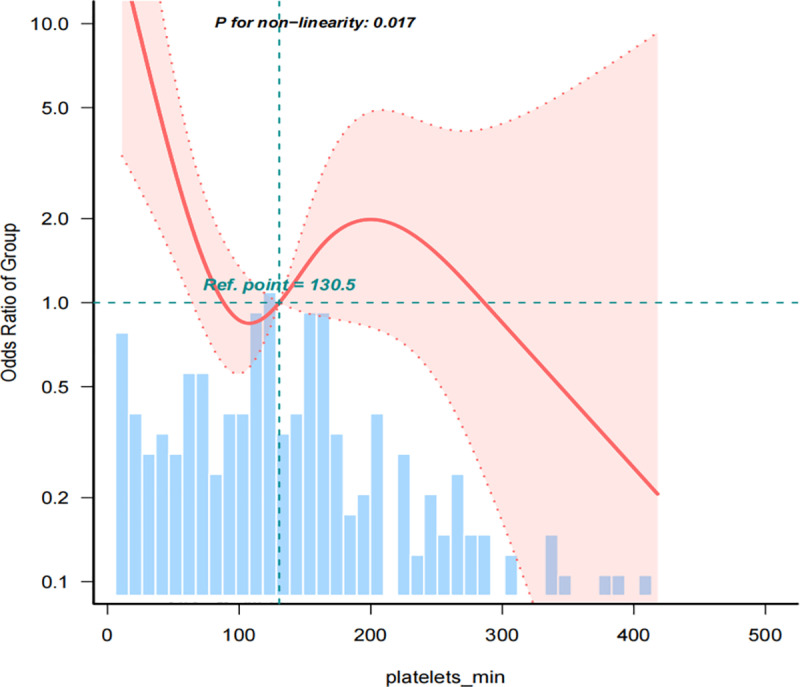
The nonlinear relationships between PLT-min and in-hospital mortality in Multiple Myeloma in ICU.

By employing a multivariate logistic regression analysis and fitting smooth curves, after adjusting for covariates in model 4, we further explore the relationship between different PLT-min levels and the risk of mortality. The fitted curve reveals a nonlinear relationship (p = 0.017), suggesting that the relationship between platelet count and mortality risk may depend on specific PLT-min ranges rather than uniformly affecting all count levels (Fig 2).

### Subgroup analysis

**Fig 3:**
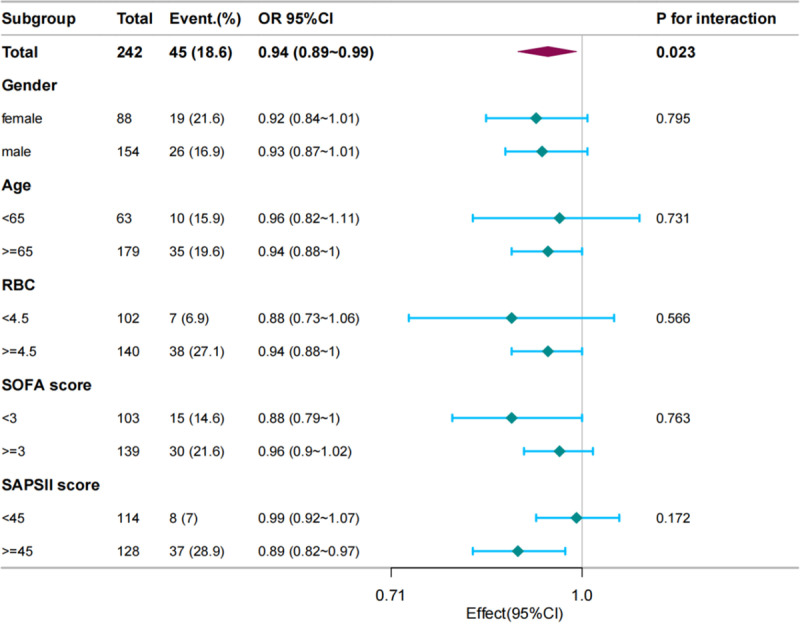
Forest Plots for the Association of PLT-min and in-hospital mortality in Multiple Myeloma in ICU.

Using the PLT-min as a continuous variable(at per 10 × 10^9^/L), subgroup analysis was conducted by gender, age, RBC-max, SOFA score, and SAPSII score to compare the mortality rates between different subgroups and the reference group. As shown in Fig 3, the p-values for the interaction among subgroups in each category were all greater than 0.05, indicating consistent effects of platelet values on mortality rates among different subgroups with no statistical differences.Additionally, subgroup analysis was performed based on gender, age, RBC, SOFA score, and SAPSII score, revealing consistent effects of the maximum platelet count on mortality rate (S1 Fig).

### Sensitivity analysis

Further conducting multivariate logistic regression analysis and fitting smooth curves using the maximum platelet count, we still observe a nonlinear relationship between PLT-max and mortality (p=0.026)(S1 Table and S2 Fig). To maximise statistical power and minimise bias that might occur if patients with missing data were excluded from analyses, we used multivariate multiple imputation with chained equations We used multiple imputation, based on 5 replications and a chained equation approach method to impute missing values [27]. We repeated all analyses with the complete data cohort for comparison. The results of simple imputation and multiple imputation are consistent: both indicate that the PLT-min is an independent risk factor for in-hospital mortality of critically ill MM patients.The S2-S4 Tables gives additional details of the statistical analyses.

## Discussion

This study revealed that the in-hospital mortality rate among ICU-admitted MM patients is approximately 18.59%. Platelet count emerges as a standalone prognostic indicator for in-hospital mortality in critically ill MM. Moreover, the study delineated a nonlinear association between platelet levels and in-hospital mortality. The pattern observed in the multifactorial logistic regression model aligns with the fitted curve. Notably, the correlation between platelet count (both minimum and maximum values during hospitalization) and mortality remains unaffected by variables such as gender, age, anemia, cardiovascular, liver, and kidney comorbidities. Cytogenetic abnormalities, peripheral blood plasma cell presence, elevated lactate dehydrogenase (LDH), renal function impairment, and extramedullary plasma cell infiltration stand out as adverse prognostic factors for MM [2,20]. While the SAPSII score primarily gauges organ failure severity, the SOFA score serves as a forecasting parameter for mortality in critically ill patients [28]. A study akin to this, investigating MM risk factors using the MIMIC database, identified serum calcium as an independent risk determinant for ICU mortality in critically ill MM patients [21]. Although univariate logistic regression revealed associations between RBC, PT, INR, calcium, SOFA and SAPSII scores, platelet count, and in-hospital mortality, data limitations in the MIMIC IV database necessitated the exclusion of LDH due to a high missing rate of 34.7%.Accounting for collinearity among variables like PT and INR, RBC and HB within the full model, demonstrates the independence of platelet count in its correlation as a continuous variable with mortality. Similarly, a trend of correlation between platelet count as a categorical variable and mortality persists. Subgroup analyses further underscore the stability of the correlation between platelet count and in-hospital mortality across various demographics and clinical scores. Robust results from statistical analysis on the highest platelet count endorse a view that reduced platelet count represents an independent risk factor for heightened in-hospital mortality in MM.

In review of 1027 patients with newly diagnosed MM, Kyle Robert A eta [15]found thrombocytopenia (PLT < 100 × 10^9^/L) was present initially in 5% of patients. Thrombocytosis (PLT ≥ 500 × 10^9^/L) was present in 2% of patients. The baseline data we collected align with our observations, indicating that not all patients with MM experience a decrease in platelet count. In fact, there is a minority of patients who exhibit an elevation in platelet count. However, this study did not thoroughly investigate the relationship between platelet levels and patient mortality. A study by Dae-Sik Kim et al. [14] suggests that a higher neutrophil-to-lymphocyte ratio (NLR), lower platelet count, and higher C-reactive protein (CRP) levels before treatment independently contribute to poorer overall survival (OS). These effects remain statistically significant regardless of age, renal function, or exposure to new drugs [14].Nevertheless, this study solely focused on the initial test data of MM patients from a single-center at the time of their initial diagnosis. The dynamic changes in platelet levels during the treatment process were not evaluated. Since this study included critically ill ICU inpatients, their condition could rapidly deteriorate or improve during treatment. Instead of solely investigating the initial platelet (PLT) count upon admission, the study extracted the lowest and highest platelet count values throughout the entire duration of hospitalization to examine their correlation with in-hospital mortality. This approach provides a more stable and comprehensive reflection of the association between PLT and mortality.

Fritz and colleagues [29] observed that the platelet half-life of MM patients was significantly shortened, with an average of 73 hours, while the platelet half-life of the healthy control group was 107 hours. They suspected that platelet activation and consumption that finally manifested in shortened platelet half-life. It is still confusing if overt thrombocytopenia develops only when the compensatory capacity of the bone marrow finally becomes exhausted. Further studies are needed to elucidate the pathophysiologic processes involved. Analysis of surface markers of platelet activation revealed significantly elevated median P-selectin and CD63 levels and Annexin V binding at diagnosis. O’Sullivan et al.’s study showed that compared to the healthy control group, the levels of P-selectin and CD63 were significantly elevated at the time of MM diagnosis and remained high after treatment; however, both pre- and post-treatment, the patient response to agonists showed significant decreases in PAC-1 binding and P-selectin levels [30]. This suggests the presence of abnormal platelet activation and reduced function. Consistent with the above study, this study also observed that platelet count and mortality rate did not have a simple linear relationship. It is speculated that abnormal platelet activation led to a shortened half-life due to impaired platelet function, while abnormal plasma cells led to reduced platelet reactivity. Bone marrow hematopoiesis may compensate for platelet deficiency by increasing platelet numbers to make up for functional defects. MM may exhibit both decreased PLT and abnormal function, and both may undergo dynamic adjustments in different bone marrow microenvironmental conditions.

The strength of this study lies in the inclusion of a relatively large sample of critically ill MM patients, not limited to a single medical institution or newly diagnosed individuals. Appropriate statistical methods were used to determine that low platelet count was an independent risk factor for in-hospital mortality, showing a nonlinear relationship between the two rather than a simple linear one. Multivariable logistic regression analyses were performed on platelet count as a continuous and categorical variable to confirm the consistency of the associations. Subgroup classification based on covariate selection also suggested a robust correlation between the two. Sensitivity analysis further demonstrated a correlation between the maximum and minimum platelet counts during hospitalization and in-hospital mortality, showing an overall trend of decreasing platelets being positively correlated with in-hospital mortality.

However, this study has several limitations: firstly, as a retrospective study based on the MIMIC IV database, the diagnoses of multiple myeloma and other comorbidities were based on administrative diagnostic codes, which might lead to errors in classification. Secondly, due to the nature of the database, the study lacked some potential variables such as cytogenetic results, bone marrow pathology results, MM staging, and some important related laboratory results (such as beta-2 microglobulin, lactate dehydrogenase), and treatment information. Severity assessment was performed using SOFA and SAPSII scores instead of assessing the severity of MM. Thirdly, there was no prospective validation in an independent cohort.

## Conclusion

In conclusion, our study indicates that decreased platelet levels in MM patients lead to an increased ICU in-hospital mortality rate, and there is a nonlinear relationship between the two. However, additional prospective cohort studies are needed to confirm the relationship between platelet levels and mortality in MM patients.

## Supporting information

S1 Fig Forest Plots for the Associations of PLT-max and in-hospital mortality in Multiple Myeloma in ICU. (TIF)

S2 Fig The nonlinear relationship between PLT-max and in-hospital mortality in Multiple Myeloma in ICU. (TIF)

S1 Table Multivariable logistic regression analyses of the association of platelet with in-hospital mortality. (DOCX)

S2 Table Baseline demographic characteristics of the study population stratified by the lowest platelet count. (DOCX)

S3 Table Association of covariates and in-hospital morality. (DOCX)

S4 Table Multivariable logistic regression analyses of the association of PLT-min with in-hospital mortality. (DOCX)
