# Supplementary figures and images for: Association between Platelet Count and In-Hospital Mortality in Critical Patients with Multiple Myeloma: A Cohort Study
Source: PLoS One. 2025 Jun 5;20(6):e0323429. doi: 10.1371/journal.pone.0323429 (PMC12140237; doi:10.1371/journal.pone.0323429)

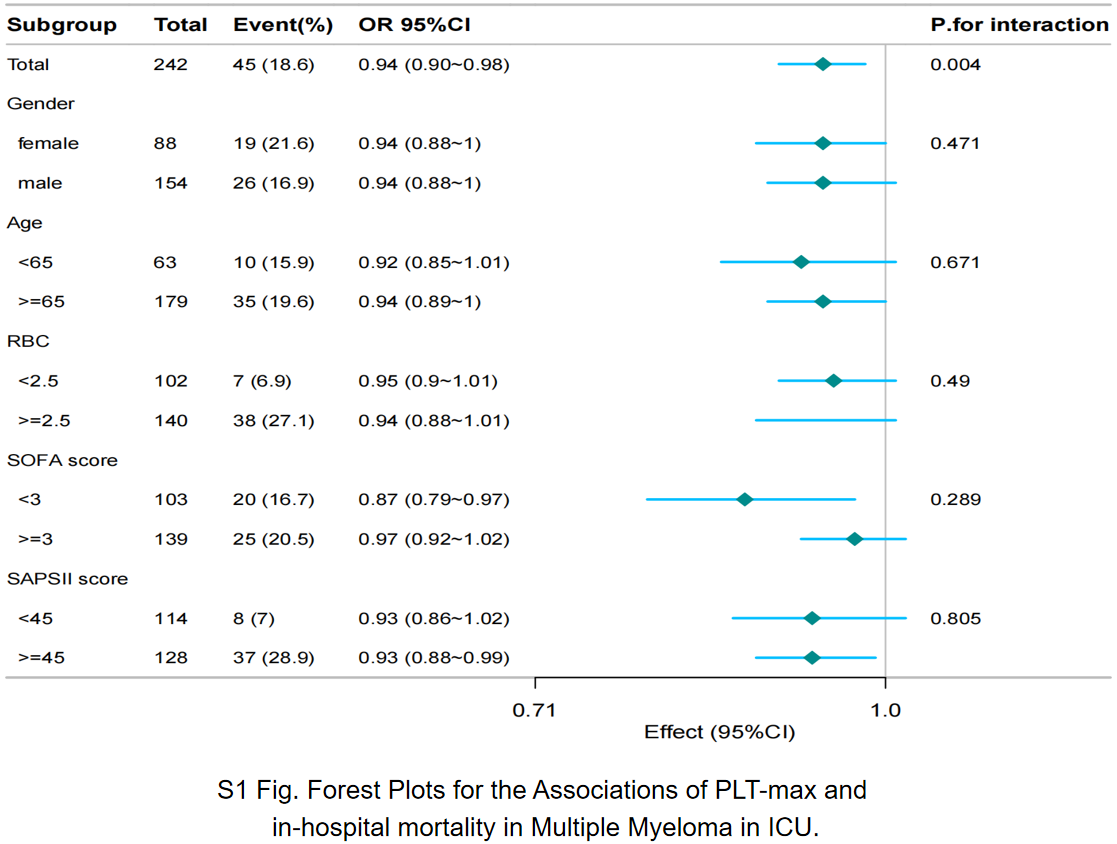

Supplement: S1 Fig — (TIF) [file pone.0323429.s001.tif]

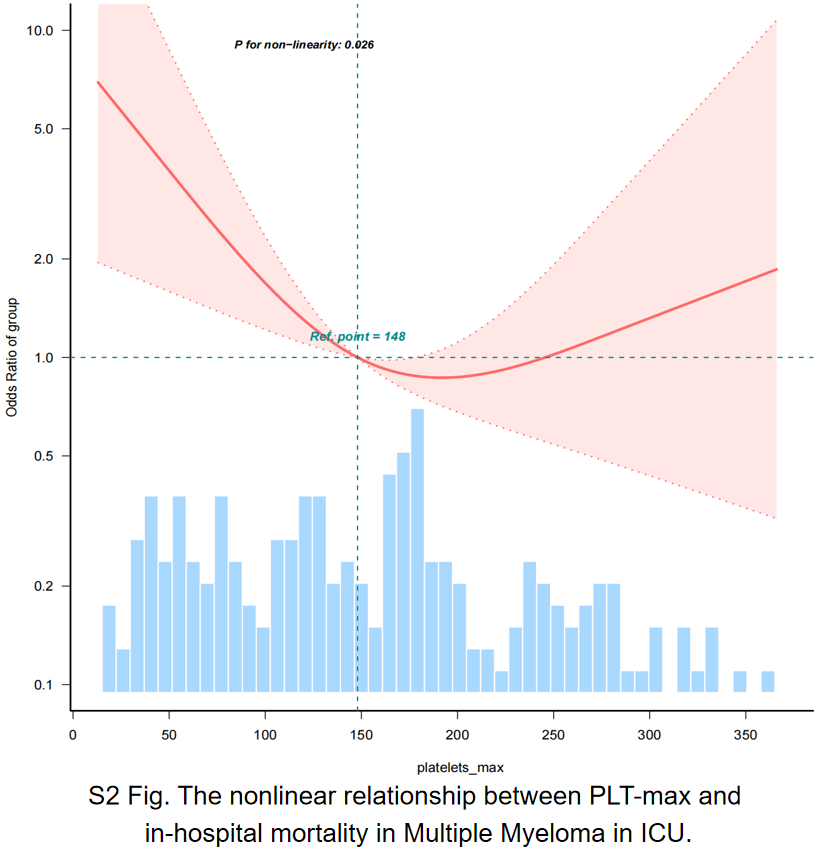

Supplement: S2 Fig — (TIF) [file pone.0323429.s002.tif]
