# Supplementary material for: Association between Platelet Count and In-Hospital Mortality in Critical Patients with Multiple Myeloma: A Cohort Study
Source: PLoS One. 2025 Jun 5;20(6):e0323429. doi: 10.1371/journal.pone.0323429 (PMC12140237; doi:10.1371/journal.pone.0323429)
Supplement: S1 Table — (DOCX) [file pone.0323429.s003.docx]

Supplementary Material 1

Multivariable logistic regression analyses of the association of platelet with in-hospital mortality

|  | Platelet-max | | Platelet-max quartiles | | | | |
| --- | --- | --- | --- | --- | --- | --- | --- |
|  | Total N=242,n.event%=45(18.6%) | | Q1(N=81);n.event%=24(29.6%) | Q2(N=80);n.event%=12(15.0%) | | Q3(N=81);n.event%=9(11.1%) | |
|  | OR(95%CI) | p | OR(95%CI) | OR(95%CI) | p | OR(95%CI) | p |
| Model1 | 0.94 (0.90-0.98) | 0.004 | 1(Ref) | 0.42 (0.19-0.91) | 0.028 | 0.30 (0.13-0.69) | 0.005 |
| Model2 | 0.93 (0.89-0.97) | 0.001 | 1(Ref) | 0.34 (0.15-0.77) | 0.009 | 0.25 (0.11-0.60) | 0.002 |
| Model3 | 0.94 (0.90-0.98) | 0.005 | 1(Ref) | 0.35 (0.14-0.83) | 0.018 | 0.19 (0.07-0.51) | 0.001 |
| Model4 | 0.95 (0.90-0.99) | 0.012 | 1(Ref) | 0.34 (0.13-0.87) | 0.024 | 0.18 (0.06-0.52) | 0.002 |

The highest platelet count was entered as continuous variable per 10×10^9^

Model1 crude model

Model2 adjusted for Gender,Age

Model3 adjusted for Model1 and RBC-min,calcium-max,INR-min

Model4 adjusted for Model2 and SOFA score,SAPSII
