# Supplementary material for: Association between Platelet Count and In-Hospital Mortality in Critical Patients with Multiple Myeloma: A Cohort Study
Source: PLoS One. 2025 Jun 5;20(6):e0323429. doi: 10.1371/journal.pone.0323429 (PMC12140237; doi:10.1371/journal.pone.0323429)
Supplement: S2 Table — (DOCX) [file pone.0323429.s004.docx]

Supplementary Material 2

Baseline demographic characteristics of the study population stratified by the lowest platelet count.

|  | Total (n = 242) | Platelet-min quartiles(*10^9^/L) | | | P value |
| --- | --- | --- | --- | --- | --- |
|  |  | Q1 (n = 81) | Q2 (n = 80) | Q3(n = 81) |  |
|  |  | <95 | 95-160 | =160 |  |
| Gender, n (%) |  |  |  |  | 0.838 |
| female | 88 (36.4) | 50 (62.5) | 53 (66.2) | 51 (62.2) |  |
| male | 154 (63.6) | 30 (37.5) | 27 (33.8) | 31 (37.8) |  |
| Age, | 72.1 ± 11.4 | 66.8 ± 11.7 | 72.1 ± 10.9 | 71.2 ± 11.9 | 0.009 |
| Mortality, n (%) | 45 (18.6) | 25 (31.2) | 8 (10) | 12 (14.6) | 0.001 |
| RBC-max(×10^12^/L) | 5.3 ± 2.1 | 5.3 ± 2.3 | 5.1 ± 2.1 | 5.3 ± 2.0 | 0.862 |
| RBC-min(×10^12^/L) | 2.6 ± 1.1 | 2.6 ± 1.1 | 2.7 ± 1.2 | 2.4 ± 1.0 | 0.436 |
| Hematocrit-min(%) | 25.9 ± 5.7 | 23.2 ± 4.1 | 26.3 ± 5.6 | 28.2 ± 6.0 | < 0.001 |
| Hematocrit-max(%) | 29.8 ± 5.6 | 27.6 ± 4.8 | 30.2 ± 4.9 | 31.5 ± 6.2 | < 0.001 |
| Hemoglobin-min(g/dL) | 8.6 ± 1.9 | 7.8 ± 1.4 | 8.6 ± 1.9 | 9.3 ± 1.9 | < 0.001 |
| Hemoglobin-max(g/dL) | 9.7 ± 1.8 | 9.2 ± 1.6 | 9.8 ± 1.6 | 10.2 ± 1.9 | 0.002 |
| Platelets-min(×10^9^/L) | 142.0 ± 106.1 | 45.3 ± 24.2 | 127.8 ± 21.1 | 250.1 ± 105.9 | < 0.001 |
| Platelets-max(×10^9^/L) | 171.6 ± 119.1 | 67.2 ± 34.1 | 157.0 ± 35.9 | 287.7 ± 123.2 | < 0.001 |
| WBC-min(×10^9^/L) | 6.0 (3.6, 9.5) | 3.3 (1.6, 6.0) | 6.3 (4.2, 8.7) | 7.8 (5.3, 11.8) | < 0.001 |
| WBC-max(×10^9^/L) | 8.1 (5.0, 12.4) | 5.0 (2.6, 9.2) | 8.2 (6.4, 12.2) | 10.3 (7.6, 15.8) | < 0.001 |
| Urea nitrogen-min | 27.5 (17.0, 46.0) | 28.0 (16.8, 48.0) | 27.5 (18.0, 39.2) | 28.0 (17.0, 46.0) | 0.989 |
| Urea nitrogen-max | 33.0 (19.2, 55.0) | 31.5 (19.0, 55.0) | 32.0 (20.8, 50.8) | 35.0 (20.2, 54.8) | 0.926 |
| Calcium-min(mg/dL) | 8.0 ± 1.1 | 7.8 ± 1.3 | 8.0 ± 1.0 | 8.2 ± 1.0 | 0.09 |
| Calcium-max(mg/dL) | 8.5 ± 1.2 | 8.3 ± 1.5 | 8.7 ± 1.0 | 8.6 ± 1.1 | 0.115 |
| Chloride-min(mmol/L) | 101.6 ± 7.2 | 103.2 ± 7.2 | 101.0 ± 7.4 | 100.7 ± 6.8 | 0.058 |
| Chloride-max(mmol/L) | 105.7 ± 6.6 | 107.4 ± 6.4 | 105.6 ± 6.2 | 104.1 ± 6.8 | 0.006 |
| Creatinine-min(mg/dL) | 1.3 (0.8, 2.5) | 1.1 (0.8, 2.1) | 1.4 (1.0, 2.4) | 1.1 (0.8, 2.6) | 0.258 |
| Creatinine-max(mg/dL) | 1.5 (1.0, 2.9) | 1.5 (1.0, 2.6) | 1.7 (1.2, 2.9) | 1.5 (0.9, 2.9) | 0.278 |
| Glucose-min(mg/dL) | 108.0 (91.0, 129.0) | 107.5 (89.0, 125.2) | 109.0 (93.0, 133.0) | 107.0 (90.2, 125.5) | 0.681 |
| Glucose-max(mg/dL) | 142.0 (116.2, 174.0) | 142.0 (117.0, 172.5) | 147.5 (121.5, 180.0) | 131.0 (110.0, 162.2) | 0.218 |
| Sodium-min(mmol/L) | 136.0 ± 6.9 | 136.2 ± 7.1 | 135.3 ± 7.9 | 136.4 ± 5.7 | 0.597 |
| Sodium-max(mmol/L) | 139.3 ± 5.3 | 139.8 ± 5.8 | 139.2 ± 5.2 | 138.9 ± 5.0 | 0.573 |
| Potassium-min(mmol/L) | 4.0 ± 0.6 | 3.9 ± 0.6 | 4.0 ± 0.6 | 4.0 ± 0.6 | 0.462 |
| Potassium-max(mmol/L) | 4.6 ± 1.0 | 4.5 ± 0.9 | 4.7 ± 1.1 | 4.7 ± 0.9 | 0.202 |
| INR-min(s) | 1.2 (1.1, 1.4) | 1.3 (1.1, 1.5) | 1.3 (1.2, 1.4) | 1.2 (1.1, 1.3) | 0.014 |
| INR-max(s) | 1.4 (1.2, 1.7) | 1.4 (1.2, 1.8) | 1.4 (1.2, 1.7) | 1.2 (1.1, 1.6) | 0.015 |
| PT-min(s) | 15.7 ± 6.4 | 15.8 ± 5.2 | 15.7 ± 5.5 | 15.7 ± 8.1 | 0.995 |
| PT-max(s) | 17.6 ± 8.3 | 17.9 ± 7.7 | 17.4 ± 6.9 | 17.4 ± 10.0 | 0.936 |
| APTT-min(s) | 28.9 (25.8, 34.4) | 29.0 (25.9, 32.7) | 28.5 (25.4, 35.1) | 28.9 (26.2, 36.1) | 0.734 |
| APTT-max(s) | 32.3 (27.6, 44.8) | 32.0 (27.8, 38.1) | 33.7 (27.7, 42.5) | 30.9 (27.5, 51.3) | 0.804 |
| Cerebrovascular disease, n (%) |  |  |  |  | 0.998 |
| No | 221 (91.3) | 73 (91.2) | 73 (91.2) | 75 (91.5) |  |
| Yes | 21 ( 8.7) | 7 (8.8) | 7 (8.8) | 7 (8.5) |  |
| Pulmonary disease, n (%) |  |  |  |  | 0.246 |
| No | 192 (79.3) | 63 (78.8) | 68 (85) | 61 (74.4) |  |
| Yes | 50 (20.7) | 17 (21.2) | 12 (15) | 21 (25.6) |  |
| Diabetes, n (%) |  |  |  |  | 0.201 |
| No | 190 (78.5) | 68 (85) | 59 (73.8) | 63 (76.8) |  |
| Yes | 52 (21.5) | 12 (15) | 21 (26.2) | 19 (23.2) |  |
| Renal disease, n (%) |  |  |  |  | 0.2 |
| No | 139 (57.4) | 52 (65) | 45 (56.2) | 42 (51.2) |  |
| Yes | 103 (42.6) | 28 (35) | 35 (43.8) | 40 (48.8) |  |
| Liver disease, n (%) |  |  |  |  | 0.082 |
| No | 236 (97.5) | 76 (95) | 78 (97.5) | 82 (100) |  |
| Yes | 6 ( 2.5) | 4 (5) | 2 (2.5) | 0 (0) |  |
| SOFA score | 4.0 ± 2.4 | 4.8 ± 2.8 | 3.6 ± 1.8 | 3.7 ± 2.2 | < 0.001 |
| SAPSII score | 49.0 ± 13.1 | 49.2 ± 12.7 | 48.7 ± 13.5 | 49.1 ± 13.3 | 0.965 |
