# Supplementary material for: Association between Platelet Count and In-Hospital Mortality in Critical Patients with Multiple Myeloma: A Cohort Study
Source: PLoS One. 2025 Jun 5;20(6):e0323429. doi: 10.1371/journal.pone.0323429 (PMC12140237; doi:10.1371/journal.pone.0323429)
Supplement: S3 Table — (DOCX) [file pone.0323429.s005.docx]

Supplementary Material 3

Association of covariates and in-hospital morality

| Variable | OR 95%CI | p |
| --- | --- | --- |
| Gender | 1.52 (0.79~2.93) | 0.214 |
| Age | 1.01 (0.98~1.04) | 0.364 |
| RBC-max | 1.06 (0.92~1.23) | 0.431 |
| RBC-min | 1.15 (0.87~1.51) | 0.325 |
| Hematocrit-min | 1 (0.95~1.06) | 0.883 |
| Hematocrit-max | 1.01 (0.95~1.07) | 0.833 |
| Hemoglobin-min | 0.97 (0.81~1.16) | 0.756 |
| Hemoglobin-max | 1.01 (0.84~1.21) | 0.916 |
| Platelets-min | 0.99 (0.99~1) | 0.004 |
| Platelets-max | 0.99 (0.99~1) | 0.003 |
| WBC-min | 1.01 (0.96~1.07) | 0.582 |
| WBC-max | 1.01 (0.97~1.05) | 0.668 |
| Urea nitrogen-min | 1.01 (1~1.02) | 0.023 |
| Urea nitrogen-max | 1.01 (1~1.02) | 0.071 |
| Calcium-min | 0.62 (0.44~0.86) | 0.005 |
| Calcium-max | 0.77 (0.55~1.07) | 0.121 |
| Chloride-min | 1 (0.95~1.04) | 0.929 |
| Chloride-max | 1.03 (0.98~1.09) | 0.187 |
| Creatinine-min | 1.05 (0.92~1.2) | 0.481 |
| Creatinine-_max | 1.05 (0.95~1.16) | 0.315 |
| Glucose-min | 1 (0.99~1.01) | 0.742 |
| Glucose-max | 1 (1~1.01) | 0.011 |
| Sodium-min | 0.97 (0.92~1.01) | 0.115 |
| Sodium-max | 1.04 (0.98~1.1) | 0.216 |
| Potassium-min | 1.3 (0.77~2.21) | 0.323 |
| Potassium-max | 1.1 (0.79~1.53) | 0.562 |
| INR-min | 1.68 (1.06~2.66) | 0.028 |
| INR-max | 1.58 (1.1~2.28) | 0.014 |
| PT-min | 1.06 (1.01~1.11) | 0.011 |
| PT-max | 1.04 (1~1.07) | 0.035 |
| APTT_min | 1.03 (1.01~1.06) | 0.004 |
| APTT_max | 1.01 (1~1.02) | 0.059 |
| Cerebrovascular-disease1 | 1.03 (0.33~3.23) | 0.956 |
| Pulmonary-disease1 | 0.66 (0.28~1.58) | 0.351 |
| Diabetes | 0.98 (0.51~1.89) | 0.959 |
| Renal disease | 0.3 (0.1~0.89) | 0.031 |
| Liver disease | 4.62 (0.9~23.69) | 0.067 |
| SOFA score | 1.12 (0.98~1.27) | 0.035 |
| SAPSII | 1.01 (0.99~1.03) | 0.361 |
