# Supplementary material for: Association between Platelet Count and In-Hospital Mortality in Critical Patients with Multiple Myeloma: A Cohort Study
Source: PLoS One. 2025 Jun 5;20(6):e0323429. doi: 10.1371/journal.pone.0323429 (PMC12140237; doi:10.1371/journal.pone.0323429)
Supplement: S4 Table — (DOCX) [file pone.0323429.s006.docx]

Supplementary Material 4

Multivariable logistic regression analyses of the association of PLT-min with in-hospital mortality

|  | Platelet-min | | Platelet-min quartiles | | | | |
| --- | --- | --- | --- | --- | --- | --- | --- |
|  | Total N=242,n.event%=45(18.6%) | | Q1(N=81);n.event%=23(28.4%) | Q2(N=80);n.event%=10(12.5%) | | Q3(N=81);n.event%=12(14.8%) | |
|  | OR(95%CI) | p | OR(95%CI) | OR(95%CI) | p | OR(95%CI) | p |
| Model1 | 0.99 (0.99-1.00) | 0.004 | 1(Ref) | 0.24 (0.11-0.58) | 0.002 | 0.38 (0.17-0.82) | 0.013 |
| Model2 | 0.99 (0.99-1.00) | 0.002 | 1(Ref) | 0.21 (0.09-0.74) | 0.001 | 0.33 (0.15-0.74) | 0.007 |
| Model3 | 0.99 (0.99-1.00) | 0.005 | 1(Ref) | 0.22 (0.09-0.56) | 0.001 | 0.35 (0.15-0.79) | 0.012 |
| Model4 | 0.94 (0.89-0.99) | 0.023 | 1(Ref) | 0.25 (0.11-0.63) | 0.004 | 0.38 (0.16-0.91) | 0.027 |
